# Proactive breast cancer risk assessment in primary care: a review based on the principles of screening

**DOI:** 10.1038/s41416-023-02145-w

**Published:** 2023-02-03

**Authors:** Juliet A. Usher-Smith, Sarah Hindmarch, David P. French, Marc Tischkowitz, Sowmiya Moorthie, Fiona M. Walter, Rebecca A. Dennison, Francisca Stutzin Donoso, Stephanie Archer, Lily Taylor, Jon Emery, Stephen Morris, Douglas F. Easton, Antonis C. Antoniou

**Affiliations:** 1 The Primary Care Unit, Department of Public Health and Primary Care, University of Cambridge, Cambridge, UK; 2 Manchester Centre for Health Psychology, Division of Psychology and Mental Health, School of Health Sciences, Faculty of Biology, Medicine and Health, University of Manchester, Manchester, UK; 3 Department of Medical Genetics, National Institute for Health Research Cambridge Biomedical Research Centre, University of Cambridge, Cambridge, UK; 4 PHG Foundation, University of Cambridge, Cambridge, UK; 5 Wolfson Institute of Population Health, Queen Mary University of London, London, UK; 6 Department of Psychology, University of Cambridge, Cambridge, UK; 7 Centre for Cancer Research and Department of General Practice, University of Melbourne, Melbourne, VIC Australia; 8 Centre for Cancer Genetic Epidemiology, Department of Public Health and Primary Care, University of Cambridge, Cambridge, UK

**Keywords:** Population screening, Disease prevention

## Abstract

In the UK, the National Institute for Health and Care Excellence (NICE) recommends that women at moderate or high risk of breast cancer be offered risk-reducing medication and enhanced breast screening/surveillance. In June 2022, NICE withdrew a statement recommending assessment of risk in primary care only when women present with concerns. This shift to the proactive assessment of risk substantially changes the role of primary care, in effect paving the way for a primary care-based screening programme to identify those at moderate or high risk of breast cancer. In this article, we review the literature surrounding proactive breast cancer risk assessment within primary care against the consolidated framework for screening. We find that risk assessment for women under 50 years currently satisfies many of the standard principles for screening. Most notably, there are large numbers of women at moderate or high risk currently unidentified, risk models exist that can identify those women with reasonable accuracy, and management options offer the opportunity to reduce breast cancer incidence and mortality in that group. However, there remain a number of uncertainties and research gaps, particularly around the programme/system requirements, that need to be addressed before these benefits can be realised.

## Background

Breast cancer is the most common cancer worldwide and accounts for ~25% of cancers and 15% of cancer deaths in women [1]. Population-based screening programmes inviting women from age 50 for mammographic screening have been shown to reduce breast cancer mortality [2].

In the UK, the National Institute for Health and Care Excellence (NICE) recommends that women at moderate or high risk of breast cancer (Table 1) be offered additional breast screening/surveillance from younger ages and risk-reducing medication. It is estimated that more than 250,000 UK women are at moderate or high risk and so eligible for such interventions [3].

**Table 1:** Risk thresholds according to NICE guidelines for lifetime risk of developing breast cancer assessed from age 20 and 10-year risk of developing breast cancer between ages 40 and 50.

	Risk thresholds			
Risk	Near-population risk	Moderate risk	High risk 1	High risk 2
Lifetime risk from age 20	<17%	17–30%	≥30%	*
10-year risk between ages 40 and 50	<3%	3–8%	≥8%	*

*The High risk 2 group includes patients with known *BRCA1, BRCA2* and *TP53* mutations and rare conditions that carry an increased risk of breast cancer such as Peutz–Jegher Syndrome (STK11), Cowden (PTEN) and familial diffuse gastric cancer (E-Cadherin). Patients who have not had genetic testing, but who have a greater than 30% probability of carrying a *BRCA1, BRCA2* or *TP53* mutation are included in this group.

Women with high-risk pathogenic genetic variants (e.g., in *BRCA 1/2*) are identified through cascade testing led through specialist genetics clinics. How women at moderate or high risk but without known high-risk genetic variants within their family are best identified is less clear. Considerable work has been done [4, 5], and is ongoing [6], around the inclusion of breast cancer risk assessment to identify these women at the time of mammographic screening. However, this will miss younger women eligible for screening and preventative options. Primary care provides a potential route through which to identify these women.

In the initial version of the current guidance [3], published in November 2019, NICE recommended that the role of primary care was limited to identifying women at moderate or high risk when they present with concerns. Research has shown that this opportunistic approach leads to only a small fraction of those at moderate or high risk being identified: in one survey among screening attendees it was estimated that 8.8% of women in their forties would be eligible for additional screening/surveillance and risk-reducing medication [7], but only 17.5% of that group had been seen in family history or clinical genetics services. Relying on women to self-present with concerns may also exacerbate existing health inequalities or disadvantage groups who have lower levels of health literacy.

In June 2022, following a stakeholder consultation, the NICE statement recommending identification of women only when they present with concerns was withdrawn to allow the proactive identification of women at increased risk of breast cancer within primary care. This shift to proactive identification substantially changes the role of primary care, in effect paving the way for a primary care-based screening programme to identify those at moderate or high risk of breast cancer. Any such programme will require establishing processes for identifying women at increased risk, pathways for offering them appropriate information and management, and robust assessments of likely benefits and costs.

In this article, we review the current literature surrounding the proactive identification of women at increased risk of breast cancer within primary care. We use the principles of the consolidated framework for screening [8] to establish whether the process meets the conditions for a screening programme and to identify key challenges, uncertainties and research gaps. Our focus is on the UK given the recent change in NICE guidance but the principles apply to the introduction of similar programmes internationally.

## Disease/condition principles

### Epidemiology of the disease/condition

Increasing age is the most common risk factor for breast cancer with incidence rates rising sharply until menopause and more slowly thereafter [9]. Other known risk factors that can be used to identify women at elevated risk include: family history of breast cancer [10], lifestyle and anthropometric factors such as alcohol consumption [11], height [12] and body mass index [13], hormone-related risk factors such as ages at menarche and menopause [14], oral contraceptive use [15] and menopausal hormone replacement therapy use [16], reproductive factors such as parity and age at first live birth [17] and breast density [18]. In addition, genetic susceptibility to breast cancer is conferred by rare pathogenic variants in high- and moderate-risk genes (including *BRCA1, BRCA2, PALB2, CHEK2, ATM, BARD1, RAD51C* and *RAD51D*) [19] and common low-penetrance alleles which combine multiplicatively and can be represented as polygenic scores (PGS) [20].

Based on distributions of known risk factors in the UK, it is estimated that approximately 17% of women are at above population-level risk and 48% of cases of breast cancers occur in that group [21]. Studies at breast cancer screening have similarly identified around 18.5% of women over age 50 at moderate or high risk [22]. In the only study in primary care, 11% of women were at above population-level risk [23]. That study had a response rate of only 16.1% though and risk was assessed using family history alone. The risk distribution based on multifactorial risk assessment in a population identified through primary care is, therefore, unknown.

### Natural history of the disease/condition

In recent years the biology of breast cancer has become better understood, including increasing knowledge of the histological and molecular characteristics and drivers for cell proliferation [24]. There is also clear evidence of benefits and opportunities for early detection, with the typical breast cancer tumour volume doubling time estimated to be 150 days [25] and 5-year survival 97.9% at Stage I and falling to 26.2% at Stage IV [26]. The relationship between the natural history of the disease and breast cancer risk is less well understood. It is known that variants in several of the high-risk genes are more strongly associated with oestrogen receptor (ER)-negative or triple-negative breast cancer, while polygenic scores are more strongly associated with ER-positive disease [20, 27]. Several lifestyle factors also show differential risks by subtype, pointing to differences in the natural history and hence potentially screening efficacy. Studies are ongoing among those women attending mammographic screening that will hopefully inform this [28].

### Target population

As described above, there is considerable work being conducted to evaluate the potential for offering risk assessment alongside mammography to identify women attending screening who are above near-population risk [4–6]. The principal target population for proactive identification in primary care is, therefore, those women who would potentially benefit from interventions but are not yet eligible to be invited for population-based screening. In the UK, this is women under 50.

Identifying this target group is straightforward using electronic healthcare records. A much greater challenge is being able to reach these women. A number of potential strategies exist. These include postal or text message invitations and invitations at the time of other healthcare delivery. All of these approaches have the potential to exacerbate existing health inequalities through low engagement. For example, in the context of breast cancer screening, uptake of risk assessment questionnaires at the time of mammography was significantly lower in areas with higher deprivation [22]; and in the only study to date to proactively seek to identify high-risk women within UK primary care, only 16.1% of women (*n* = 1127/7012) returned family history questionnaires [23], with those from minority ethnic communities and those with low literacy underrepresented. More active strategies for engagement, including tailored approaches with underrepresented populations, will therefore be needed [29]. These might include working with patient navigators or community health champions alongside text message primers, computer prompts, behaviourally informed messaging and targeted telephone outreach that have been associated with greater uptake of NHS Health Checks within primary care [30].

## Test/intervention principles

### Screening test performance characteristics

In the context of breast cancer risk assessment, the test is the estimation of an individual’s risk of breast cancer. Several risk prediction models exist. Table 2 summarises the risk factors included in some of the most recently developed and widely used models [21, 31–35].

**Table 2:** Risk factors included in some of the most recently developed and widely used models and currently available tools to support risk calculation.

	BCSC [33]	BOADICEA [21]	Gail [34]	Tyrer–Cuzick/IBIS [31]	iCARE [32]	QCancer10 [36]
Genetic/familial risk factors						
Family history	•	•	•	•	•	•
Rare pathogenic variants in moderate- and high-risk susceptibility genes		•		•		
Polygenic risk score	•	•		•	•	
Demographic factors						
Country of origin (country-specific cancer incidence)		•				
			•			
Birth cohort/decade of birth		•				
Ashkenazi Jewish ancestry		•		•		
Ethnicity (other than European/Ashkenazi Jewish)	•					•
Lifestyle factors						
Body mass index		•		•	•	
Height		•		•	•	
Alcohol use		•			•	•
Hormonal/reproductive factors						
Age at menarche		•	•	•	•	
Oral contraceptive use		•			•	•
Age at menopause		•			•	
Menopausal hormone therapy		•		•	•	•
Number of live births		•		•	•	
Age at first live birth		•	•	•	•	
Other factors						
Mammographic density	•	•		•		
History of hyperplasia/benign disease			•	•	•	•
History of lobular carcinoma in situ				•		
Previous breast biopsy	•		•	•		
Manic depression or schizophrenia						•
Prior lung, blood or ovarian cancer						•
Tools to support risk calculation						
Online tool	tools.bcsc-scc.org/AdvBC6yearRisk/#/	canrisk.org/	bcrisktool.cancer.gov/	ibis-risk-calculator.magview.com/		qcancer.org/10yr/female/
Software package for integration within electronic healthcare records						•

*BCSC* Breast Cancer Surveillance Consortium, *BOADICEA* Breast and Ovarian Analysis of Disease Incidence and Carrier Estimation Algorithm, *iCARE* Individualised Coherent Absolute Risk Estimators.

It is important that risk prediction models are well calibrated (i.e., the predicted risks agree with the observed risks within different risk categories), and that they have good discriminatory ability for distinguishing between those who will develop the disease and unaffected women in the target population. Studies assessing the performance of these models have consistently shown that model performance is maximised when the joint effects of risk factors are considered in multifactorial breast cancer risk prediction [21, 36, 37]. Population-based studies of multifactorial risk models that include polygenic scores in pre-menopausal women or those under the age of 50 have shown that the BOADICEA and Tyrer–Cuzick models have moderate-to-good discrimination with AUCs or c-indices of 0.68–0.69 [38, 39] and the iCARE model an AUC of 0.64. BOADICEA and iCARE are also well calibrated with a ratio of expected to observe the number of cases (E/O) of 0.97 [39] and 0.9 [36], respectively, while the Tyrer–Cuzick model shows evidence of overestimating risk amongst those in the highest-risk decile (E/O: 1.54) [39].

The benefits of multifactorial risk assessment are particularly seen when considering the effects on risk classification. For example, a population aged 40 with unknown family history would without risk assessment be considered at near-population risk. Using the full set of risk factors within the BOADICEA model, 86.4% of women in the population would be classified at near-population risk of developing breast cancer and 13.6% at moderate or high risk. Using questionnaire-based risk factors alone would classify only 1.8% of those women at moderate risk and none as high risk [21]. The absolute numbers, however, differ between risk models [40]. A further major limitation at present of these risk models, particularly those incorporating PGS, is that to date they have all been developed using data primarily from individuals of European ancestry. As a result, they perform less well in other ancestral groups [41] and have the potential to increase inequalities in care. Further research to develop or adapt risk models for use within other ancestral groups is needed before breast cancer risk assessment can be offered to the population at large.

Whichever risk prediction model is chosen, obtaining accurate estimates requires accurate data on relevant risk factors. Many of the risk factors included in multifactorial risk models, particularly those relating to genetic/familial risk and reproductive factors, are not routinely available within primary care. With the exception of the QCancer10 model, all the risk models in Table 2 require additional data collection. Mechanisms to collect this additional data are, therefore, needed. Several tools have been designed to support this [42–46]. Each varies in the time they take to complete due to variations in the complexity of the family history required and different user interfaces. Models including genetic risk factors also require biological samples. Currently, almost all germline cancer genetic testing in the NHS is done using blood samples. However, saliva is an equally good alternative for most testing and has the advantage of allowing the sample to be taken at home, making it more acceptable for women [47], and is more straightforward to transport. Incorporating breast density is more challenging in this age group as most women will not have had breast imaging. Research is ongoing to define the magnitude of breast cancer risk associated with mammographic density in young women and the acceptability of low-dose mammography for risk assessment in this group [48]. However, this introduces significant additional challenges in terms of clinical workflow and the service model.

### Interpretation of screening test results

There are existing well-defined, evidence-based and agreed risk thresholds that are set by NICE (Table 1) [3]. The categorisation of women following risk assessment is, therefore, straightforward. The challenge is communicating the results of the risk assessment and how these risks relate to recommended management options in such a way that they are clearly interpretable and support informed decision-making. As highlighted in a recent review [49], this is not straightforward as risk is a multidimensional concept, with lay perceptions often being resistant to change, dominated by family history and appraised differently from how healthcare professionals communicate risk. Developing ways to explain breast cancer risk information in a way that is intelligible to the general population is therefore important. Tailored communication strategies may be needed for those with low literacy. Ideally, these would be based on evidence-based approaches to communicating risk [50] and tested in the specific context of breast cancer risk assessment in primary care.

### Post-screening test options

The post-screening management options are already well-defined by NICE guidelines [3]. All women, independent of risk, should receive lifestyle advice, with all those at moderate or high risk of breast cancer offered risk-reducing medication and enhanced breast cancer screening/surveillance dependent on age and risk level (Table 3). Women at very high risk due to known genetic variants are also eligible for risk-reducing surgery. Currently, most very high-risk women will be identified through cascade testing within secondary care, meaning that risk-reducing medication and enhanced screening/surveillance are the two main management options for women identified through primary care.

**Table 3:** Enhanced screening for those women under age 50 identified as at moderate or high risk of breast cancer (reproduced from NICE guidelines [3]).

	Moderate risk	High risk				
Age	Moderate risk of breast cancer	High risk of breast cancer (with a 30% or lower probability of a BRCA or TP53 mutation)	Untested but greater than 30% BRCA carrier probability	Known BRCA1 or BRCA2 mutation	Untested by greater than 30% TP53 carrier probability	Known TP53 mutation
20–29					Annual MRI	Annual MRI
30–39		Consider annual mammography	Annual MRI and consider annual mammography	Annual MRI and consider annual mammography	Annual MRI	Annual MRI
40–49	Annual mammography	Annual mammography	Annual MRI and annual mammography	Annual MRI and annual mammography	Annual MRI	Annual MRI

The evidence for enhanced screening/surveillance among these women largely comes from population-based trials offering women aged 40–49 earlier mammography. From these trials, it is estimated that annual mammography would reduce mortality from breast cancer by 12–29%, with a 0.1% risk of over-diagnosis, a false-positive recall every 10 years and fatal radiation-induced breast cancer on average once every 76,000–97,000 years [51]. There is also low-quality evidence to suggest that mammographic screening specifically in women aged 50 years or less with a family history of breast cancer reduces mortality and that invasive breast cancers diagnosed in the screened group are significantly smaller than those diagnosed in unscreened women of similar age and less likely to have spread to lymph nodes [52, 53].

There is high-quality evidence that tamoxifen reduces breast cancer incidence in pre-menopausal women who do not have a diagnosis of breast cancer [54, 55]. Based on NICE eligibility and assuming a 25% uptake, over 50 years it is estimated that 11 cases of breast cancer could be avoided per 1000 women who are offered risk-reducing medication [3]. Tamoxifen has been associated with a small increase in the risk of endometrial cancer (RR 2.13; 95% CI, 1.36–3.32) and venous thromboembolisms (RR 1.93; 95% CI, 1.41–2.64) [55]. However, the risk of venous thromboembolisms is largely confined to the active treatment period [54] and, where sub-group analyses have been performed, significant increases in the risk of endometrial cancer have not been seen in women under 50 [56].

## Programme/system principles

### Screening programme infrastructure

As with the introduction of any new service within primary care, the introduction of a proactive breast cancer risk assessment would require additional funding to account for the additional time for the work undertaken by primary care [57–61]. This is particularly important given the recent increasing demands on primary care. In the UK, potential routes for such funding include the Quality and Outcomes Framework, Directed Enhanced Services and central models of payment as used for the NHS Health Check Programme.

There is also a need for risk assessment tools that are integrated within the electronic health records to enable collection, recording and sharing of risk information between patients, professionals and care settings [45, 61–63] and streamlining of the collection of risk factor information through the use of patient-facing tools. Of the available breast cancer risk assessment tools, only the QCancer10 model [35] is currently available as a software development kit to build it within electronic health records but this does not incorporate many of the well-established breast cancer risk factors. Other risk prediction models rely on external web-based applications that require primary care professionals to enter data manually, substantially adding to the time required for conducting risk assessments. Primary care is leading the way in terms of implementing interoperable electronic health records within the UK and this has been further expedited by the COVID-19 pandemic. However, the challenges of integrating new risk assessment tools into the NHS [64], and primary care specifically [65], are likely to remain and are made more complex by how, currently, IT digital services are commissioned, adopted and maintained through individual local Integrated Care Systems rather than nationally.

Alongside these digital tools, additional training for primary care healthcare professionals will be required if discussions around multifactorial risk are to take place in primary care. Previous studies have consistently identified primary care providers’ lack of knowledge about breast cancer risk assessment, particularly genetic testing, and lack of confidence and access to referral guidance as significant barriers to engagement [45, 61, 63, 66–69]. Initiatives to provide such training are ongoing and include resources across relevant NHS and professional colleges websites [70, 71]. Evaluation of these training resources is needed.

More generally, there will also need to be updates to the current referral pathways for those at moderate or high risk of breast cancer, and sufficient capacity and funding within the wider healthcare system to provide additional screening/surveillance and consultations to discuss risk-reducing medication for those women identified as being at moderate or high risk. Providing this additional screening/surveillance will likely require the commissioning of new services: a survey of all cancer genetics leads covering 22 regional centres in the UK in 2019 showed that 15 years after the original NICE guidance was published, 10.4 million women (16.4% of the total population) were still living in regions not supplying appropriate moderate-risk surveillance and up to 42% of the population were living in areas not covered by the recommended high-risk surveillance [72]. There will also need to be sufficient laboratory capacity to process saliva or blood samples for polygenic risk score assessment and gene panel testing. As the NHS Genomic Medicine Service covers the whole range of genetic tests (not only cancer), any change would need to be modelled to assess the potential impact on other services.

### Screening programme co-ordination and integration

One of the biggest challenges raised by healthcare professionals in a focus group study of incorporating cancer risk within primary care was finding the best time and place for risk assessment [61]. Several studies in the US have shown that risk assessment is feasible in primary care waiting rooms prior to annual health check or new patient visits [73–76]. However, until genetic testing is widespread within mainstream healthcare settings, this does not allow for genetic testing to be incorporated in the risk assessment prior to discussion with the primary care professional. In a recent interview study exploring the barriers and facilitators to implementing CanRisk in UK general practice, GPs and practice nurses cited appointments around the prescription/review of contraception and hormone replacement therapy as being potentially the most appropriate opportunities, due to standard discussion around the impact of these medications on breast cancer risk [paper in preparation: SA, FSD, Carver, Yue, Cunningham, Ficorella, MT, DFE, ACA, JE, JUS, FMW. Exploring the barriers and facilitators of implementing CanRisk in primary care: a qualitative thematic framework analysis]. Some suggested that a risk assessment could also be conducted or mentioned during cervical cancer screening appointments, but there were concerns about adding additional stress and/or anxiety to these already challenging healthcare appointments. There is also evidence that women prefer information by email or online rather than during GP consultations [47]. Research evaluating different implementation strategies and the impact on primary care and uptake is needed.

It is also unclear how frequently breast cancer risk assessment should be repeated. The current NICE guidance suggests reassessment if a woman’s family history changes [3]. However, with the inclusion of modifiable lifestyle and hormonal factors in a risk assessment, changes in these risk factors, or the development of more predictive PGS over time, could potentially result in reclassification of woman’s risk category. How any such changes in risk would be handled also needs to be considered, particularly for women moving from a higher-risk category to a lower-risk category.

### Screening programme acceptability and ethics

A recent systematic review showed that primary care providers are likely to accept an increased role in breast cancer risk assessment as they already collect family history information regularly and readily perceive it as their responsibility [63]. However, if the risk assessment were to be conducted within a consultation there are concerns about how that may change the dynamic of the consultation [45] and about the depth and intrusive nature of some of the questions required for risk assessment, particularly around pregnancy and baby loss. Other concerns include the ethical justification of discussing breast cancer risk if little can be done by the woman to reduce her risk [57, 77], as well as concerns about the accuracy of risk estimates derived from models, particularly when information about risk factors is incomplete [45, 76]. The provision of training targeted at increasing knowledge about risk management strategies and risk calculation would address these concerns.

Healthcare professionals have also identified a number of ethical implications that would need to be considered in any future programme [66]. These include potential issues around autonomy, distributive justice, privacy, stigma, storage and access to genetic material and information, discrimination from employers and healthcare systems or insurers and the potential to increase inequalities as a result of the differential performance of polygenic risk scores in non-European ancestries described above.

Breast cancer risk assessment itself has been shown to be widely acceptable to the public [78–85]. Women are optimistic about receiving individualised breast cancer risk estimates, with studies reporting that 85% women consider risk assessment to be a good idea [86] and most are comfortable providing the information for the risk assessment, whether personal information, a blood sample, or a mammogram [79, 80]. Importantly, no women found to be high risk in one study regretted finding out their risk [87] and all appear to understand the principles/purpose of risk stratification [84]. The acceptability of the risk assessment taking place within primary care is limited to two studies in the US [75, 88]. These studies suggest that risk assessment, without genetic risk factors, is both acceptable and feasible to the public in the primary care context. The potential acceptability of incorporating polygenic risk scores has been demonstrated in a recent study reporting 84% uptake of a primary care-based polygenic risk assessment for colorectal cancer [89]. Additionally, evidence suggests that a risk assessment endorsed and performed by a familiar individual or institution, such as general practice, may be more acceptable to women and that uptake may be improved by easier access [83].

As the main benefit from risk assessment arises through those women who are identified as being at moderate or high risk being offered, and subsequently taking up, earlier screening/surveillance and/or risk-reducing medication, the acceptability of these management options is key. Numerous studies have demonstrated widespread reticence by primary care practitioners to discuss and prescribe risk-reducing medication for women at high risk of breast cancer [63, 90–92]. Lack of familiarity with and knowledge of preventive therapy has been identified as a contributing factor [91]. In addition, primary care providers do not perceive the initiation of risk-reducing medication as their responsibility [90]. However, general practitioners have reported greater willingness to continue a prescription if it has been started in secondary care [92]. This suggests that primary care would be more accepting of involvement in prescribing risk-reducing medication if shared-care agreements were in place.

The acceptability of risk-reducing medication amongst women is also generally low, with uptake outside trial settings only 8.7% [93]. A number of factors have been shown to contribute to poor uptake and compliance, including knowledge of the harms and benefits, and side effects [94–97]. Decisions are also influenced by age, socioeconomic status, race and ethnicity, family history, education, numeracy, psychological well-being, risk level, communication format and trust in healthcare professionals [98–100]. Access to tailored patient education materials to support informed decisions will, therefore, be needed [63].

In contrast, the offer of enhanced screening/surveillance appears to be generally acceptable to women, with one study amongst women attending familial cancer clinics reporting that 77% of women aged 30–39 and 96% aged 40–49 were vigilant with respect to mammography recommendations [101]. There are, however, persistent inequalities in access, and uptake of enhanced screening/surveillance policies for women from ethnic minorities and deprived areas who are at moderate and high risk of developing breast cancer is lower [72, 102]. There is little research exploring reasons for this and no studies recruiting women from primary care.

### Screening programme benefits and harms

The main rationale for breast cancer risk assessment is the offer of enhanced screening/surveillance and/or risk-reducing medications for women who are at increased risk but currently unaware of this. As described above, increased screening/surveillance or uptake of cancer-preventing drugs in this group would be expected to lead to reductions in cancer incidence, cancer-related morbidity and mortality. No studies, however, have been conducted in primary care to assess the impact of risk assessment on these “downstream” benefits [103] or the potential harms of over-diagnosis and overtreatment. To detect such effects would require a very large trial or observational study, in line with the generally small effects that population-based cancer screening has on these outcomes [2].

In addition to these “downstream” benefits, the risk assessment process itself potentially also has more “upstream” benefits and harms. These include the impact on future uptake of national screening, change in health-related behaviours and psychological outcomes. Quantifying these more “upstream” effects is important as any potential reduction in cancer incidence for a small proportion of the population needs to be balanced against the potential for possible net harms among the majority who will be at near-population risk, particularly if uptake of risk-reducing interventions is low amongst those at moderate or high risk.

No studies have been conducted in primary care to assess the impact of breast cancer risk assessment on these more “upstream” effects. The best available evidence comes from national screening programmes. In this setting, women in receipt of risk estimates are no less likely to attend future rounds of screening [104], while women who are told they are at high risk are considerably more likely to attend future rounds of screening [105]. This is in line with the intended purpose of identifying women at higher risk, and provides direct evidence that these intended benefits are likely to be accrued. There is also some evidence that women in receipt of estimates of higher breast cancer risk are more willing to take up, and persist with, interventions to promote healthier behaviours [106]. However, there is now good direct evidence that the provision of breast cancer risk estimates does not have a major impact on health-related behaviours such as increased physical activity or healthier eating at a population level [107]. These findings are in line with systematic reviews of the wider literature, which suggest limited population-level effects of personalised risk information [108] or personalised information derived from genetic sources [109].

With respect to psychological outcomes, although there are no data from primary care or in the age group targeted for proactive risk assessment in primary care, there are now considerable data on the effects of providing estimates of breast cancer risk to women aged 47–73 recruited via the NHS Breast Screening Programme. These data indicate that women who were told they were at higher risk were more worried about cancer, but there was no evidence of an overall effect on worry or adverse effects on anxiety [107]. Similarly, there was no evidence of low-risk estimates producing false reassurance [107], in line with systematic review evidence that concerns about false reassurance following screening test results is unfounded [110]. There is a need to show no such adverse effects when setting up a new programme with younger women. The impact of being identified as at higher risk has potentially greater implications for younger women when considering, for example, plans for a family. It is also important to note that there is a complete absence of evidence on the impact of providing risk estimates on some other outcomes, such as informed decision-making [111]. This is especially important as promoting informed decision-making surrounding screening decisions is an explicit aim of most screening programmes.

### Economic evaluation of the screening programme

A number of analyses have assessed the cost-effectiveness of earlier mammography breast cancer screening in high-risk groups. These have shown that biennial screening from age 40 onwards in women at increased risk of breast cancer is cost-effective [112] and results in a similar ratio of false-positive findings and life-years gained as biennial screening in average-risk women between age 50 and 74 [113], and having a lower starting age for women at higher risk would increase screening benefit and reduce over-diagnosis, but at a relatively higher increase in false-positive findings [114, 115].

No full cost-effectiveness analysis has been conducted on risk-reducing medication in pre-menopausal women. A cost-consequences analysis performed by the NICE guideline development panel estimated that offering risk-reducing medication in accordance with the recommendations would be cost-effective at a threshold of £20,000 per QALY if there was a gain of at least 1.71 QALYs per 1000 eligible women. In women over 50, it is estimated that 11 cases of breast cancer could be avoided per 1000 women who are offered risk-reducing medication and so the panel were confident that the costs of preventing a case of breast cancer were likely to be considered acceptable from an NHS perspective. The costs in women under 50 are not known.

To our knowledge, no economic evaluation has been conducted of proactively offering breast cancer risk assessment within primary care.

### Screening programme quality and performance management

If proactive risk assessment for breast cancer risk were to be implemented on a large scale there would need to be a clear specification and set of quality assurance standards and resources for delivering and monitoring the programme. This would include protocols for who should be tested, requirements for specimens and laboratory analysis of relevant risk assessment parameters (e.g., polygenic risk scores), and clinical follow-up of those identified as moderate or high risk, as well as expected internal quality control and performance criteria and published laboratory and clinical guidelines and standards. Quality assurance will also be needed with respect to the data used in risk assessment. This includes clinical accreditation of any discrete tests such as genotyping to obtain a polygenic score, and any risk tools or models used for this purpose.

Developing such a programme would also require dialogue with policymakers to determine what parameters are important to monitor for quality assurance and who would be responsible for the programme oversight. Given the existence of mechanisms for monitoring existing breast cancer screening programmes, these can be used as a starting point to achieve alignment with any existing standards and guidance.

## Conclusion

Proactive breast cancer risk assessment within primary care for women under 50 years currently satisfies many of the key principles of screening. Most notably, there are large numbers of women at moderate or high risk currently unidentified, there are risk models available that are able to identify those women with reasonable accuracy, and management options exist that offer the opportunity to reduce breast cancer incidence, morbidity and mortality in that group. However, there remain a number of uncertainties and research gaps that need to be addressed before these benefits can be realised (Table 4). These include the evaluation of different implementation strategies, including how to best reach women without exacerbating health inequalities, research to develop or adapt risk models for non-European groups and to develop and evaluate evidence-based approaches to communicating risk and the benefits and harms of management options to women, trials to assess the outcomes of risk assessment among women identified through primary care, and economic evaluations.

**Table 4:** Summary of current and required evidence for proactive breast cancer risk assessment in primary care mapped against the consolidated framework for screening.

Screening principle	Application to proactive breast cancer risk assessment in primary care
Disease/condition principles	
1. Epidemiology of the disease/ condition	• Breast cancer is the most common cancer worldwide and risk factors for breast cancer are well-established. • The risk distribution in a population identified from primary care is not known but it is estimated that ~17–19% of women are at above population-level risk and 48% of breast cancers occur in that group. • The majority of those women in the UK are unaware that they are at higher risk.
2. Natural history of the disease/condition	• The biology of breast cancer is reasonably well understood but it is not known if the natural history differs with breast cancer risk.
3. Target population	• The target population is women under 50. • Identifying these women is straightforward using electronic health records. • The challenge is reaching those women without exacerbating existing health inequalities. • A number of potential strategies exist but few have been tested in primary care, and data on the uptake of breast cancer risk assessment in this setting is limited.
Test/intervention principles	
4. Screening test performance characteristics	• In the context of breast cancer risk assessment, the test is the estimation of an individual’s risk of breast cancer. • Several multifactorial risk models exist. They have moderate-to-good discrimination and calibration in population-based studies in women under 50 and are better at classifying women than family history or questionnaire-based risk factors alone. • A major limitation at present is that all these risk models have all been developed using data from individuals of European ancestry. • Further research to develop or adapt risk models for other ethnic groups is needed.
5. Interpretation of screening test results	• There are existing well-defined, evidence-based and agreed risk thresholds that are set by NICE. • The challenge is communicating the results of the risk assessment so that they are understood by women. • This will require developing evidence-based approaches to communicating risk and testing those in this context.
6. Post-screening test options	• The post-screening management options for women at moderate or high risk are already well-defined by NICE guidelines. • There is high-quality evidence that tamoxifen reduces breast cancer incidence in pre-menopausal women and evidence that enhanced screening reduces disease-specific mortality and invasive breast cancer is diagnosed when smaller and less likely to have spread to lymph nodes.
Programme/system principles	
7. Screening programme infrastructure	Introduction of a proactive programme would require: • funding; • risk assessment tools that are integrated within the electronic health records; • training for primary care healthcare professionals; • updates to current referral pathways; and • commissioning of new services to ensure there is capacity within the wider healthcare system to provide additional screening and consultations to discuss risk-reducing medication for women at moderate or high risk.
8. Screening programme co-ordination and integration	• It is not known how best to integrate proactive breast cancer risk assessment within primary care. • Evaluation of different implementation strategies and the impact on primary care and uptake is needed. • It is also not known how frequently breast cancer risk assessment should be repeated.
9. Screening programme acceptability and ethics	• While primary care providers are likely to accept an increased role in breast cancer risk assessment, they have raised concerns about the nature of the risk assessment, the accuracy of risk estimates and a range of ethical implications that would need to be addressed. • There is also widespread reticence among primary care providers to discuss and prescribe risk-reducing medication, particularly without shared-care agreements. • Breast cancer risk assessment itself is widely acceptable to the public but acceptability within primary care is limited to two studies in the US. • Uptake of risk-reducing medication in women identified as at moderate or high risk is generally low. • In contrast, the offer of enhanced screening/surveillance appears to be generally acceptable to women.
10. Screening programme benefits and harms	• No studies have been conducted in primary care to assess the overall benefits and harms. • Detecting reductions in cancer incidence and cancer-related morbidity would require a very large trial. • Studies within national screening programmes suggest that women at higher risk may be more worried about cancer and are more likely to attend future screening and adopt healthier behaviours while there are no effects at population level and no evidence of low-risk estimates producing false reassurance. • Studies are needed to assess these outcomes following risk assessment among younger women and within primary care.
11. Economic evaluation of the screening programme	• Estimates suggest offering earlier screening and risk-reducing medication for those at moderate or high risk is cost-effective. • No economic evaluation has been conducted of proactively offering breast cancer risk assessment within primary care.
12. Screening programme quality and performance management	• Introduction of proactive risk assessment would require clear specification and resources for delivering and monitoring the programme, as well as a set of quality assurance standards and clinical accreditation of the risk models used. All these are yet to be developed.

## Data Availability

Not applicable.
